# Improvement of Storage Medium for Cultured Human Retinal Pigment Epithelial Cells Using Factorial Design

**DOI:** 10.1038/s41598-018-24121-8

**Published:** 2018-04-09

**Authors:** L. Pasovic, T. P. Utheim, S. Reppe, A. Z. Khan, C. J. Jackson, B. Thiede, J. P. Berg, E. B. Messelt, J. R. Eidet

**Affiliations:** 1 Department of Medical Biochemistry, Oslo University Hospital, Oslo, Norway; 2 Institute of Clinical Medicine, University of Oslo, Oslo, Norway; 3 Department of Surgery, Akershus University Hospital, Lørenskog, Norway; 4 Department of Plastic and Reconstructive Surgery, Oslo University Hospital, Oslo, Norway; 5 Department of Ophthalmology, Oslo University Hospital, Oslo, Norway; 6 Department of Ophthalmology, Stavanger University Hospital, Stavanger, Norway; 7 Department of Clinical Medicine, Faculty of Medicine, University of Bergen, Bergen, Norway; 8 Department of Oral Biology, Faculty of Dentistry, University of Oslo, Oslo, Norway; 9 Department of Biosciences, University of Oslo, Oslo, Norway

## Abstract

Storage of human retinal pigment epithelium (hRPE) can contribute to the advancement of cell-based RPE replacement therapies. The present study aimed to improve the quality of stored hRPE cultures by identifying storage medium additives that, alone or in combination, contribute to enhancing cell viability while preserving morphology and phenotype. hRPE cells were cultured in the presence of the silk protein sericin until pigmentation. Cells were then stored for 10 days in storage medium plus sericin and either one of 46 different additives. Individual effects of each additive on cell viability were assessed using epifluorescence microscopy. Factorial design identified promising additive combinations by extrapolating their individual effects. Supplementing the storage medium with sericin combined with adenosine, L-ascorbic acid and allopurinol resulted in the highest cell viability (98.6 ± 0.5%) after storage for three days, as measured by epifluorescence microscopy. Flow cytometry validated the findings. Proteomics identified 61 upregulated and 65 downregulated proteins in this storage group compared to the unstored control. Transmission electron microscopy demonstrated the presence of melanosomes after storage in the optimized medium. We conclude that the combination of adenosine, L-ascorbic acid, allopurinol and sericin in minimal essential medium preserves RPE pigmentation while maintaining cell viability during storage.

## Introduction

Age-related macular degeneration (AMD) is a leading cause of blindness in the developed world and is characterized by impairment and loss of the retinal pigment epithelium (RPE)^1^. Due to the lack of treatment options for the dry type of AMD, which affects 85% of patients, replacement of the RPE has been proposed as a future therapy for this disease^2–11^. Expectations for the application of RPE transplants to treat retinal diseases are high, and several studies have shown that this approach can restore subretinal anatomy and improve visual function^2^. A recent review by Nommiste *et al*.^12^ covers several proof-of-principle studies investigating the efficacy of different cell sources and transplantation techniques. The RPE cells used in these studies are derived from primary human stem cells, induced pluripotent stem cells and several other sources, and have been transplanted to the subretinal space by means of suspensions, strips or patches on coated polymers^13,14^. While the strategies for cell replacement are improving, the production of cell sheets that fulfill the requirements for transplantation is complex^15^ and will likely lead to centralization of specialized culture laboratories. The ability to store RPE successfully is necessary in order to transport the tissue from the culture laboratories to the transplantation clinics and make widespread use of RPE replacement therapies possible^12^. An established storage method would not only allow for transportation, but also make quality control and microbiological testing of the tissue possible^16^. With continued improvement of RPE tissue engineering approaches, and more than 20 million patients suffering from AMD worldwide^17^, an upcoming need for improved storage and transportation methods for cultured RPE is anticipated. An above-freezing temperature storage system as suggested by our research group circumvents the need for cryoprotectants, which are known to inflict freezing injury to tissues at both high and low cooling rates^18–20^.

After testing nine different storage temperatures between 4 °C and 37 °C, we found that hRPE cultures stored at 4 °C in a storage medium containing 4-(2-hydroxyethyl)-1-piperazineethanesulfonic acid (HEPES)- and sodium bicarbonate-buffered Minimum Essential Medium (MEM) preserved the greatest number of viable cells (unpublished data). An earlier study showed that the addition of 1% sericin to the cell culture medium enhanced hRPE cell maturation, most notably by increasing cell pigmentation^21^. The MEM storage medium is a defined basal medium that mainly consists of inorganic salts, vitamins and glucose. We therefore investigated the effects of supplementing this medium with many different additives, including sericin to preserve the differentiated state of the cells. The effects of the 46 individual supplements on viability of hRPE cell cultures were analyzed after ten days of storage at 4 °C. Some additives were selected based on their known or proposed effects on viability or antioxidant function in cultures of RPE or other cell types^21–32^, while others were chosen based on effects demonstrated in pilot experiments. Most of the additives have, to our knowledge, never been tested in the current setting. The effects of a total of 32 different combinations of the five most promising additives were simulated using a factorial design experiment. The single best combination of additives was selected for further study by additional experiments to assess its effects on phenotype and morphology.

## Results

### Effect of Individual and Combined Storage Medium Additives on Viability of hRPE

**Figure 1:**
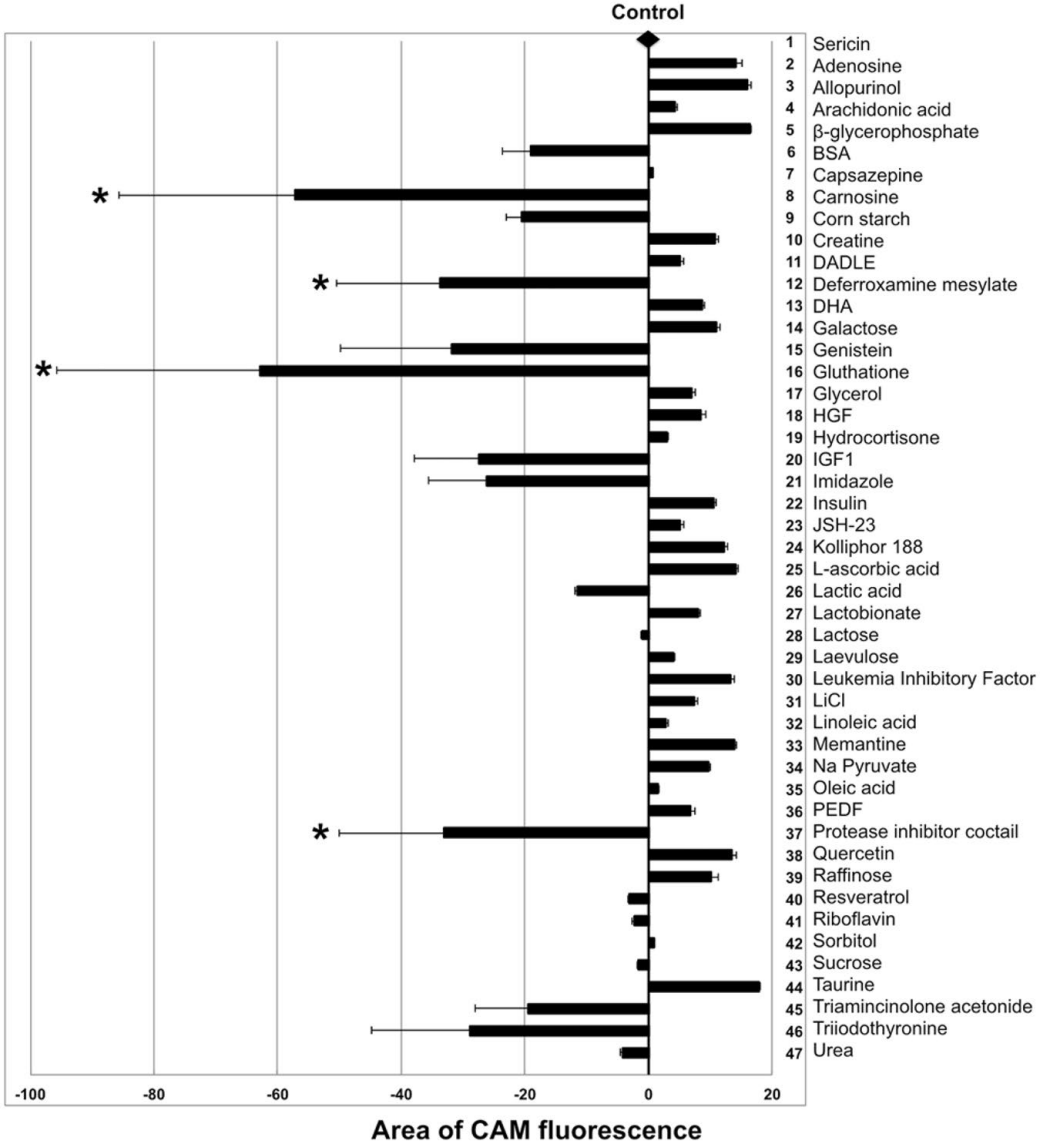
Cell viability after ten days of storage as measured by area of CAM fluorescence. The control line drawn from the black diamond represents the area of calcein-acetoxymethyl ester (CAM) fluorescence obtained in the control group (N = 18), where 1% sericin was added to the MEM-based storage medium. Other bar points are representations of CAM area fluorescence for each additive (N = 3) supplemented to MEM in the presence of 1% sericin. Resulting effects are displayed as either increasing or decreasing CAM area fluorescence compared to the control line. The addition of carnosine, deferroxamine mesylate, glutathione or the protease inhibitor cocktail to the storage medium significantly reduces cell viability as measured by CAM area fluorescence (*, *P* < 0.05). Error bars represent the standard deviation of mean values. BSA: bovine serum albumin; DADLE: [D-Ala^2^, D-Leu^5^]-Enkephalin; DHA: docosahexaenoic acid; HGF: hepatocyte growth factor; IGF1: insulin-like growth factor 1; JSH-23: 4-methyl-1-N-(3-phenylpropyl)benzene-1,2-diamine; PEDF: pigment epithelium-derived factor.

hRPE were seeded in complete EpiCM on Nunclon Δ surface plates and cultured for two days before replacing EpiCM with modified DMEM (hereafter named ≪differentiation medium≫) containing 1% sericin for 14 days. The cells had then developed pigmentation as demonstrated earlier^21^, and were stored for 10 days in storage medium plus sericin and either one of 46 different additives. The control group, containing sericin, was stored without additional additives. Cell survival following 10 days of storage in all 47 experimental groups (N = 3) was assessed by calcein-acetoxymethyl ester (CAM) fluorescence using ImageJ software to measure the culture well area covered by CAM-stained live hRPE cells. The results are presented in Fig. 1. Cells stored in MEM containing 1% sericin served as the control. Control cells covered 73.5 ± 22.3% of the culture well area. In comparison, cells that had not been stored covered 99.2 ± 0.1% of the culture well area. No single storage medium additive contributed to increasing the CAM-stained culture well area significantly compared to the control. One-way ANOVA revealed that two additives significantly reduced cell viability (carnosine and glutathione), while the Student’s t-test revealed that four additives significantly reduced cell viability (carnosine, glutathione, deferroxamine mesylate and protease inhibitor cocktail) compared to the control.

**Figure 2:**
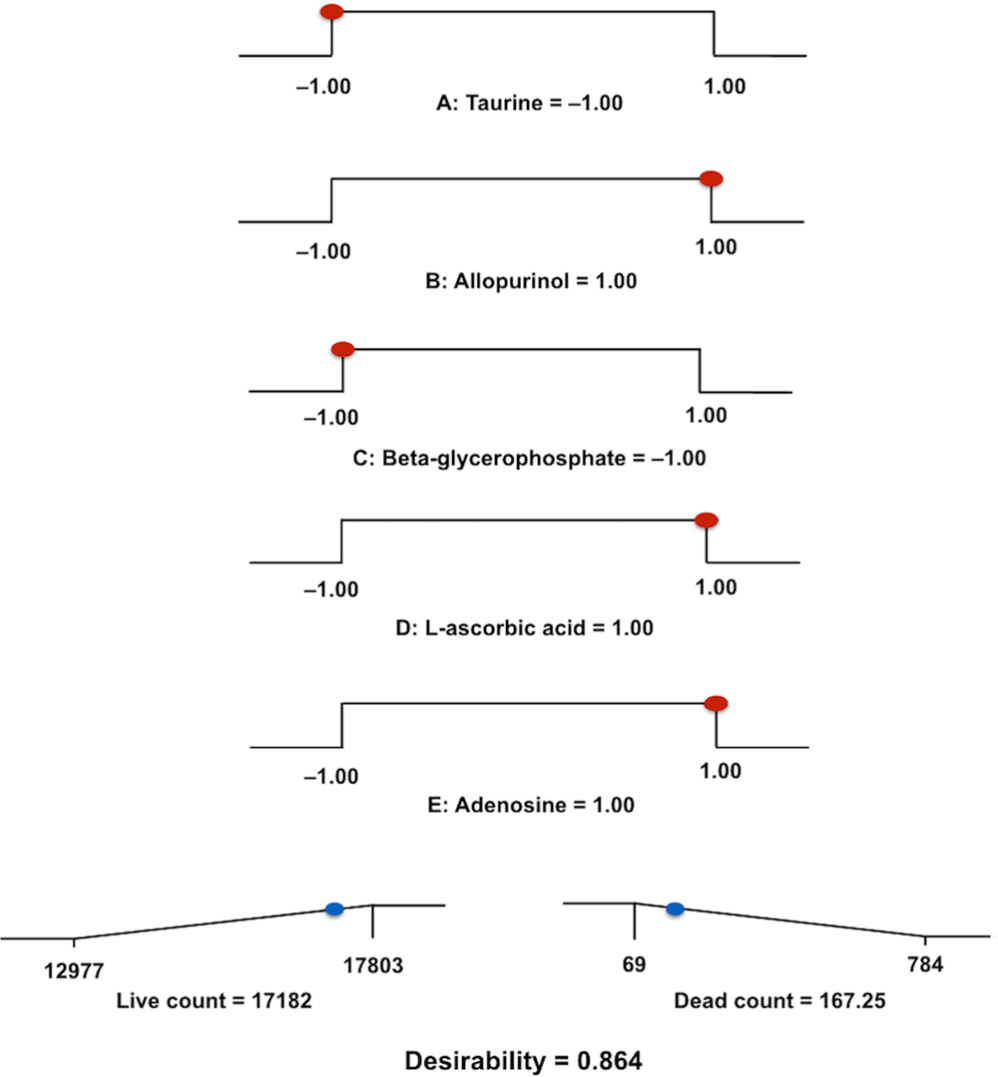
Factorial design analysis. Factorial design analysis of the five most promising additives providing a ramp display showing individual graphs for each additive in the most desirable storage medium combination. Presence of additive was set as “1”, while absence of additive was set as “−1”. The dot on each ramp represents the factor setting or response prediction for the resulting combination.

**Figure 3:**
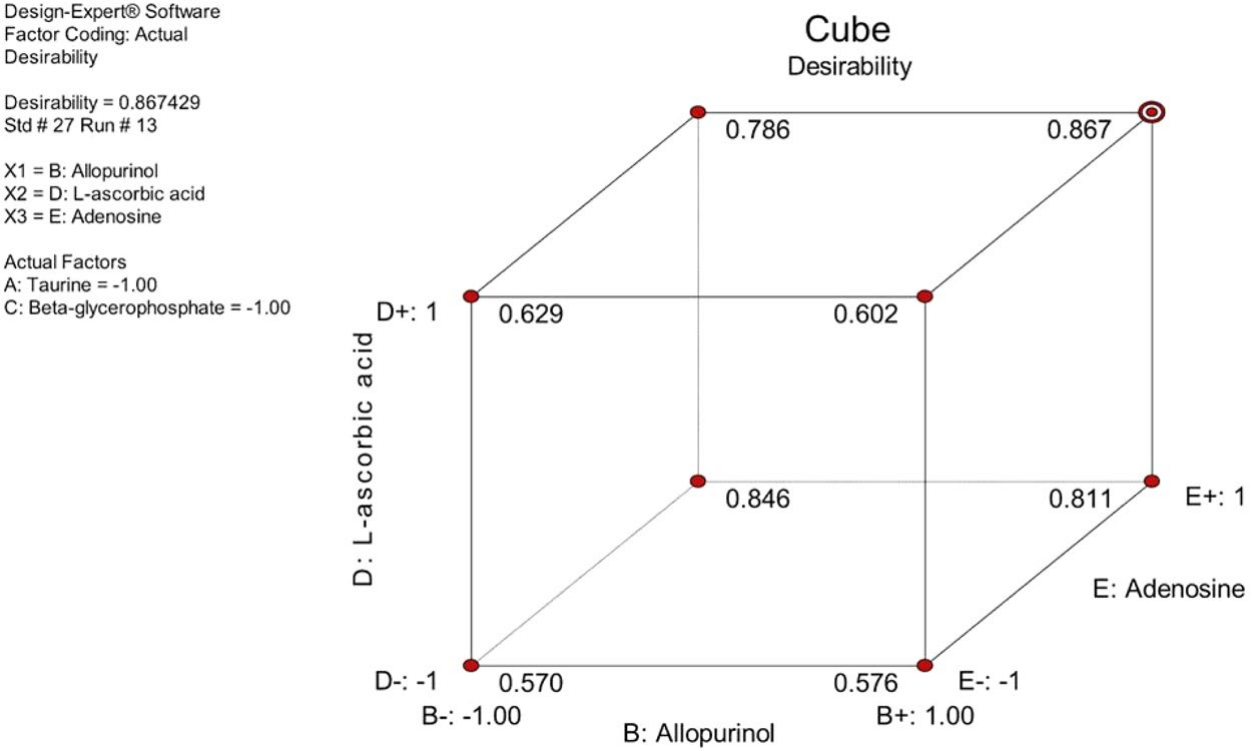
Cube plot illustrating the predicted response as a function of the three additives that created the most desirable effect. The plot shows how three factors (B, D, E) combine to affect the response. All values are predicted. Maximum desirability is reached at settings B+ , D+ and E+ (allopurinol, L-ascorbic acid and adenosine).

To investigate whether combinations of additives could increase cell viability further, the five additives which provided the largest CAM fluorescence area (adenosine, allopurinol, β-glycerophosphate, L-ascorbic acid and taurine) were selected for factorial design experiments. Normality of the data was confirmed by Design-Expert, as was absence of significant outliers on residuals plots. The data on percentage of cell viability was then power transformed as recommended by the Design-Expert software before subsequent analysis. A significant model including all possible additive combinations was computed by Design-Expert software (Stat-Ease) using ANOVA (*P* = 0.047). No single additive supplemented individually to the storage medium had a significant impact on cell viability in the factorial design experiments. The combined effects of sericin, adenosine, allopurinol and L-ascorbic acid, however, provided the highest desirability regarding both CAM-stained culture well area and number of dead cells following storage (Figs 2 and 3).

**Figure 4:**
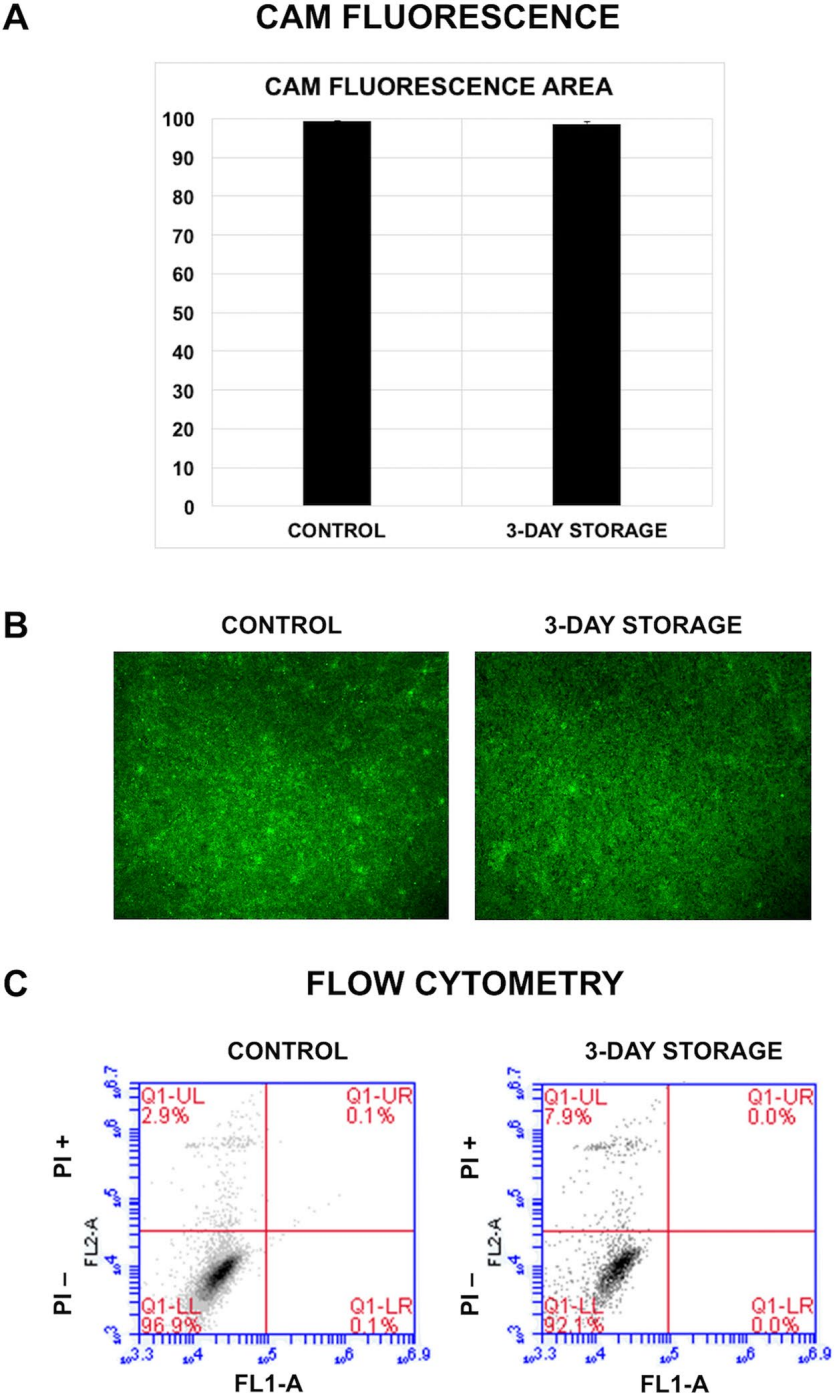
Viability of hRPE stored in the optimal combination of storage medium additives for three days. hRPE were analyzed by (**A**,**B**) quantitative fluorescence and (**C**) flow cytometry. (**A**) Cell viability as measured by area of calcein-acetoxymethyl ester (CAM) fluorescence, demonstrating similar results between groups (N = 6). Error bars represent the standard deviation of mean values. (**B**) Representative photomicrographs demonstrating similar CAM labeling between groups. (**C**) Representative flow cytometry plots of dead cells by propidium iodide exclusion in control cells and cells stored for three days (N = 3). The plots demonstrate a relatively low cell death rate in the stored group.

To confirm factorial design results, viability analysis using CAM area calculation was employed for the optimal additive combination (MEM supplemented with sericin, adenosine, allopurinol and L-ascorbic acid). hRPE cells (N = 3) were cultured at 37 °C and stored for three days at 4 °C in the optimal additive combination before being compared to control cells (N = 6) that had not been stored. Viability was similar between the groups, with a mean CAM fluorescence area of 99.2 ± 0.1% for control cells and 98.6 ± 0.5% for stored cells, respectively (Fig. 4A,B).

### Validation of Viability Data Using Flow Cytometry

The viability of hRPE stored in the optimal mix (MEM supplemented with sericin, adenosine, allopurinol and L-ascorbic acid) for three days was validated using flow cytometry with propidium iodide (PI) staining. PI passes through permeable cell membranes of dead cells and stains double-stranded DNA. PI bound to 3.1 ± 0.5% of control cells and 7.8 ± 2.5% of stored cells (*P* < 0.05), yielding a viability of 96.8 ± 0.5% and 92.1 ± 2.5%, respectively (Fig. 4C). While the difference was statistically significant (*P* = 0.03), these results support the CAM fluorescence area viability data showing only a small change in cell loss in cultures stored using the optimal combination of additives.

### pH Measurement

pH of the storage medium was assessed using pH indicator paper and demonstrated pH in the physiological area (pH = 7.4).

### Morphology of Optimal Combination hRPE

**Figure 5:**
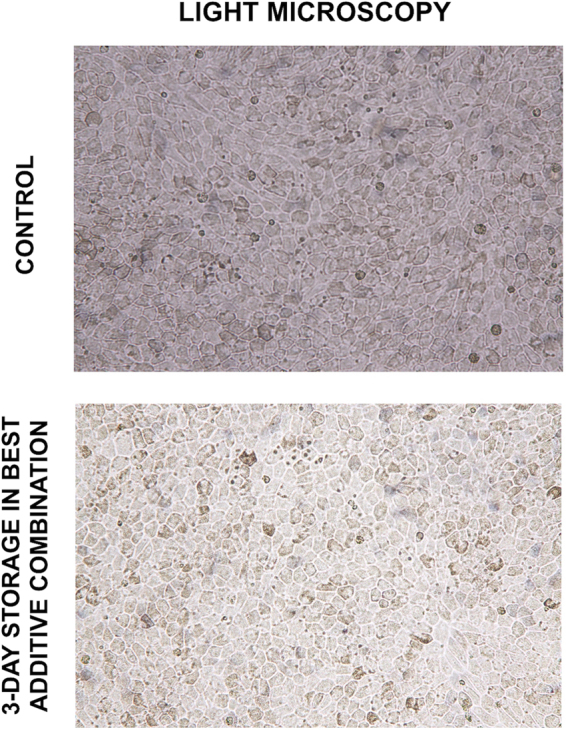
Representative sample from light microscopy observations before and after three days of storage using the best additive combination. The photomicrographs reflect the presence of melanized hRPE cells in both groups and show the classic hexagonal distribution of mature hRPE monolayers.

Both light microscopy, scanning electron microscopy and transmission electron microscopy were performed to investigate the effect of the optimal combination of storage medium additives on the morphology of hRPE. Control cells were cultured to confluence and obtained the characteristic morphology comprising a hexagonal cell shape and cytoplasmic pigmentation (Fig. 5). The same features were observed in hRPE cells that had been stored for three days using the optimal combination of storage medium additives, indicating that hRPE can be stored in this additive combination while retaining a classic RPE morphology.

**Figure 6:**
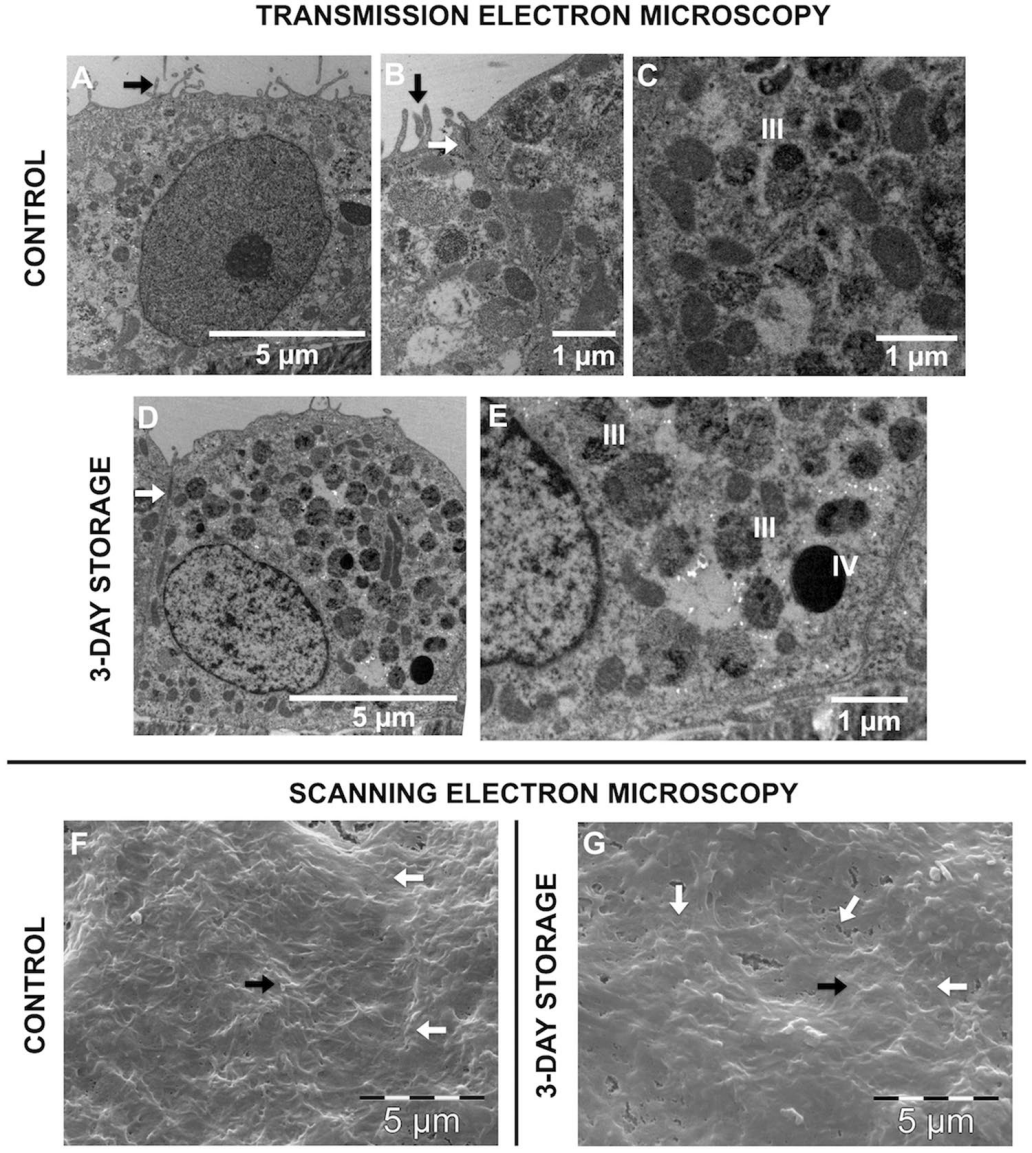
Ultrastructure of stored hRPE cells compared to control. Transmission and scanning electron microscope photomicrographs of control cells that have not been stored (**A**–**C**,**F**) and hRPE cells that have been stored at 4 °C in the optimal additive combination for three days (**D**,**E**,**G**). Intercellular tight junctions are present both between control cells and between cells stored in the optimal additive combination (white arrows in **B**,**D**). Microvilli are present in both groups, demonstrated both by transmission and scanning electron microscopy (black arrows in **A**,**B**,**F**,**G**). Photomicrographs **C** and **E** demonstrate the presence of melanosomes at stage III (some melanin pigment deposited onto internal striations) in the control group and stage III and IV (fully melanized) in the storage group. Cell borders are indicated by white arrows (**F**,**G**).

Transmission electron microscopy demonstrated that the degree of melanization in cells stored in the optimal additive combination maintained or even exceeded that of control cells (Fig. 6A–E), thereby supporting the findings made by light microscopy. Intercellular tight junctions were present both between control cells and between cells stored in the optimal additive combination (Fig. 6B,D). Microvilli were demonstrated in both groups, both by transmission and scanning electron microscopy (Fig. 6A,B,F,G).

### Proteomic Analysis of hRPE Using the Optimal Additive Combination

**Figure 7:**
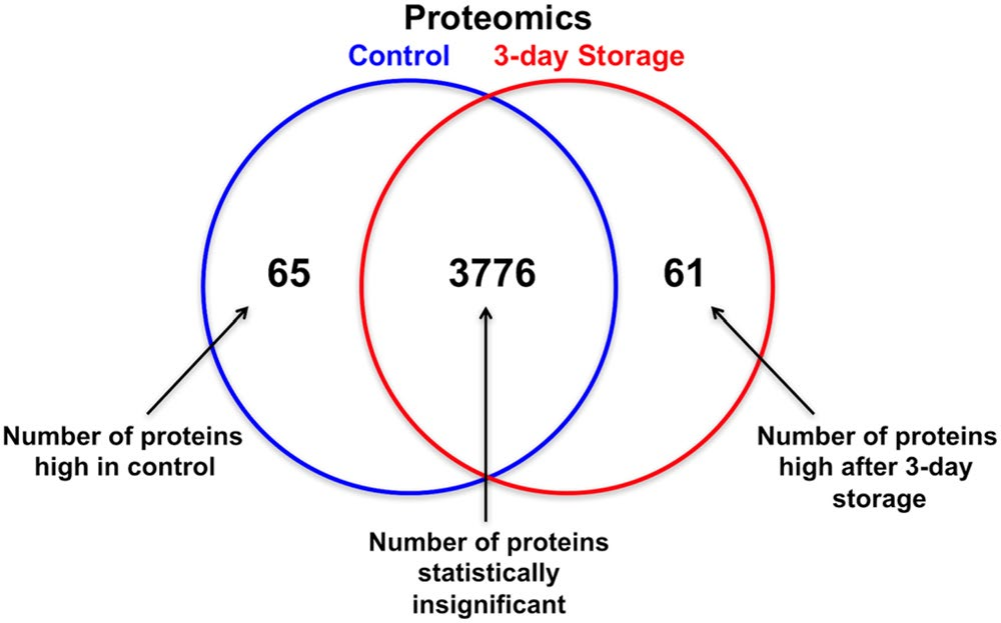
Protein expression in experimental groups. Venn diagram showing the distribution of proteins that are highly expressed in control cells, stored cells, and those which are statistically insignificant.

**Figure 8:**
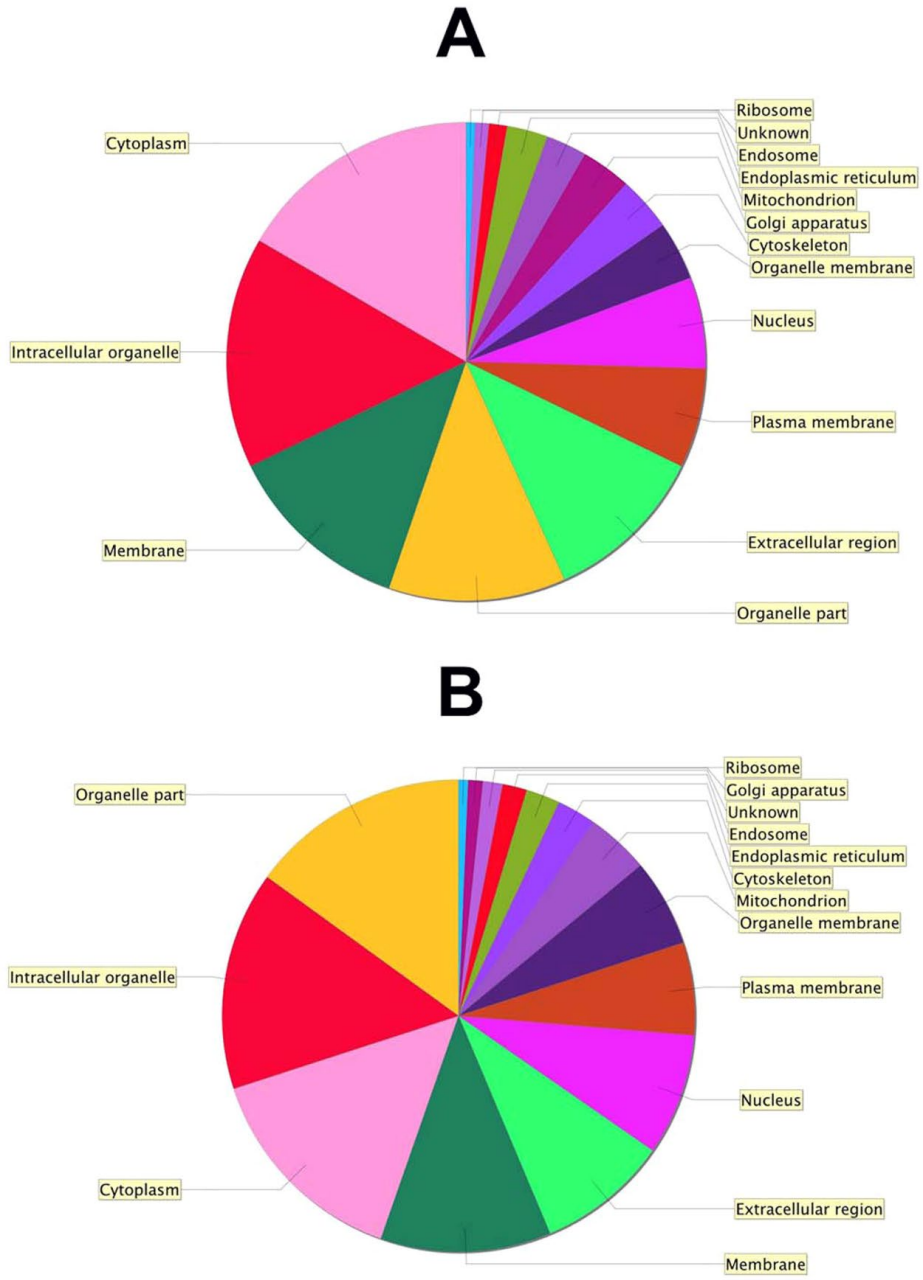
Distribution of protein functions. Gene ontology pie chart showing the distribution of protein functions in hRPE before (**A**) and after (**B**) storage according to their molecular functions as determined using Scaffold software with NCBI annotations.

Proteomic analysis was performed to investigate the effect of the optimal combination of storage medium additives on the hRPE proteome. hRPE cells stored in the optimal storage medium combination were compared to control cells that had not been stored. Of 3902 identified proteins, 126 were differentially expressed applying t-test with *P* < 0.05 (Tables 1 and 2). A total of 65 proteins (1.7%) were downregulated during storage for three days in the optimal additive mix, while 61 proteins (1.6%) were upregulated during storage in the optimal additive mix (Fig. 7). The distribution of differentially expressed proteins was similar between the groups (Fig. 8).

**Table 1:** Significantly upregulated proteins during storage (low in control cells).

Gene Symbol	Gene Description	Biological function
ACOX1	Peroxisomal acyl-coenzyme A oxidase 1	Desaturation of acyl-CoAs to 2-trans-enoyl-CoAs
ALDH18A1	Delta-1-pyrroline-5-carboxylate synthase	Synthesis of proline, ornithine and arginine
AP2A1	AP-2 complex subunit alpha-1	Component of the adaptor protein complex 2; clathrin-dependent endocytosis
ARHGAP1	Rho GTPase-activating protein 1	GTPase activator for Rho, Rac and Cdc42
ARMT1	Protein-glutamate O-methyltransferase	Formation of gamma-glutamyl methyl ester residues
BCLAF1	Bcl-2-associated transcription factor 1	Death-promoting transcriptional repressor
DAZAP1	DAZ-associated protein 1	RNA-binding protein; possibly required in spermatogenesis
DBI	Acyl-CoA-binding protein	Possible intracellular carrier of acyl-CoA esters
DBT	Lipoamide acyltransferase component of branched-chain alpha-keto acid dehydrogenase complex, mitochondrial	Conversion of alpha-keto acids to acyl-CoA and CO_2_
DDX23	Probable ATP-dependent RNA helicase DDX23	Pre-mRNA splicing
DNAJC3	DnaJ homolog subfamily C member 3	Unfolded protein response during endoplasmic reticulum stress
DSP	Desmoplakin	Anchoring of intermediate filaments to desmosomes
EIF6	Eukaryotic translation initiation factor 6	Prevents the association of the 60S ribosomal subunit with the 40S subunit
EPM2AIP1	EPM2A-interacting protein 1	Unknown
EZR	Ezrin	Connection of cytoskeletal structures to the plasma membrane; formation of microvilli
FARSB	Phenylalanine–tRNA ligase beta subunit	Regulatory tRNA ligase beta subunit
FBN2	Fibrillin-2	Component of extracellular calcium-binding microfibrils; regulation of elastic fibers
FLOT1	Flotillin-1	Possible scaffolding protein within caveolar membranes; formation of caveolae
FUCA2	Plasma alpha-L-fucosidase	Hydrolyzation of glycoproteins
GATM	Glycine amidinotransferase, mitochondrial	Synthesis of creatine precursor guanidinoacetate
GDAP2	Ganglioside-induced differentiation-associated protein 2	Unknown
HIST1H4A	Histone H4	Core nucleosome component
HNRNPD	Heterogeneous nuclear ribonucleoprotein D0	RNA-binding protein
HNRNPH1	Heterogeneous nuclear ribonucleoprotein H	Pre-mRNA processing
HNRNPM	Heterogeneous nuclear ribonucleoprotein M	Pre-mRNA binding protein
HSD17B2	Estradiol 17-beta-dehydrogenase 2	Interconversion of testosterone and androstenedione; estradiol and estrone
KIAA1468	LisH domain and HEAT repeat-containing protein KIAA1468	Unknown
KTN1	Kinectin	Kinesin-driven vesicle motility
LRPPRC	Leucine-rich PPR motif-containing protein, mitochondrial	Nuclear and mitochondrial RNA metabolism
LRRC8A	Volume-regulated anion channel subunit LRRC8A	Essential component of the volume-regulated anion channel
LRSAM1	E3 ubiquitin-protein ligase LRSAM1	Regulation of signaling pathways, cell adhesion, self-ubiquitylation, and cargo sorting during receptor endocytosis
MRPL28	39S ribosomal protein L28, mitochondrial	Component of the 39S mitochondrial ribosome subunit
MRRF	Ribosome-recycling factor, mitochondrial	Release of ribosomes from mRNA
MYH9	Myosin-9	Cytokinesis, cell shape, secretion
MYO7A	Unconventional myosin-VIIa	Intracellular movements
NUDT19	Nucleoside diphosphate-linked moiety X motif 19	Hydrolysis various CoA esters
NUP155	Nuclear pore complex protein Nup155	Component of the nuclear pore complex
PDHB	Pyruvate dehydrogenase E1 component subunit beta, mitochondrial	Conversion of pyruvate to acetyl-CoA and CO_2_
POLR2E	DNA-directed RNA polymerases I, II, and III subunit RPABC1	Subunit of RNA polymerase II
PRCP	Lysosomal Pro-X carboxypeptidase	Cleavage of C-terminal amino acids
PRDX2	Peroxiredoxin-2	Involved in redox regulation of the cell
PRDX3	Thioredoxin-dependent peroxide reductase, mitochondrial	Involved in redox regulation of the cell
PRKCSH	Glucosidase 2 subunit beta	Beta-subunit of glucosidase II, an ER glycan-processing enzyme
PRKDC	DNA-dependent protein kinase catalytic subunit	DNA double-strand break repair
PRPF8	Pre-mRNA-processing-splicing factor 8	Assembly of spliceosomal proteins
PRPS2	Ribose-phosphate pyrophosphokinase 2	Synthesis of phosphoribosylpyrophosphate (PRPP), essential for nucleotide synthesis
PTCD3	Pentatricopeptide repeat domain-containing protein 3, mitochondrial	Mitochondrial RNA-binding protein
PTPRA	Receptor-type tyrosine-protein phosphatase alpha	Regulation of integrin signaling, cell adhesion and proliferation
RAB7A	Ras-related protein Rab-7a	Key regulator in endo-lysosomal trafficking
RPL18	60S ribosomal protein L18	Component of the ribosomal 60S subunit
RPL37A	60S ribosomal protein L37a	Component of the ribosomal 60S subunit
SLC25A3	Phosphate carrier protein, mitochondrial	Transport of phosphate groups from the cytosol to the mitochondrial matrix
SOSTDC1	Sclerostin domain-containing protein 1	Bone morphogenetic protein antagonist
SRP54	Signal recognition particle 54 kDa protein	Transfer of presecretory protein from ribosomes to TRAM (translocating chain-associating membrane protein)
STAG2	Cohesin subunit SA-2	Component of the cohesin complex
SUCLG2	Succinyl-CoA ligase [GDP-forming] subunit beta, mitochondrial	Citric acid cycle
SULT1A1	Sulfotransferase 1A1	Sulfate conjugation of catecholamines, phenolic drugs and neurotransmitters
TOR1A	Torsin-1A	Protein folding, processing, stability and localization
TRA2B	Transformer-2 protein homolog beta	Pre-mRNA splicing
UBA1	Ubiquitin-like modifier-activating enzyme 1	Ubiquitin conjugation
VPS18	Vacuolar protein sorting-associated protein 18 homolog	Vesicle-mediated protein trafficking to lysosomal compartments

**Table 2:** Significantly downregulated proteins during storage (high in control cells).

Gene Symbol	Gene Description	Biological function
ABCA1	ATP-binding cassette sub-family A member 1	Transmembrane transport
ATP1A1	Sodium/potassium-transporting ATPase subunit alpha-1	Hydrolysis of ATP coupled with the exchange of sodium and potassium ions across the plasma membrane
ATP1A3	Sodium/potassium-transporting ATPase subunit alpha-3	Hydrolysis of ATP coupled with the exchange of sodium and potassium ions across the plasma membrane
ATP6V1C2	V-type proton ATPase subunit C 2	Subunit of the vacuolar ATPase
CD2AP	CD2-associated protein	Adapter protein between membrane proteins and the actin cytoskeleton
COASY	Bifunctional coenzyme A synthase	CoA biosynthetic pathway
COPZ1	Coatomer subunit zeta-1	Binds dilysine motifs, reversibly associates with Golgi non-clathrin-coated vesicles
CTTN	Src substrate cortactin	Organization of the actin cytoskeleton
EIF2S2	Eukaryotic translation initiation factor 2 subunit 2	Early protein synthesis
EIF4A3	Eukaryotic initiation factor 4A-III	ATP-dependent RNA helicase
FAH	Fumarylacetoacetase	Tyrosine catabolism
FAM234A	Protein ITFG3/Protein FAM234A	Unknown
FERMT2	Fermitin family homolog 2	Scaffolding protein, activates integrin
FNDC3A	Fibronectin type-III domain-containing protein 3A	Spermatid-Sertoli adhesion in spermatogenesis
G3BP2	Ras GTPase-activating protein-binding protein 2	Probable scaffold protein, may be involved in mRNA transport
GDI2	Rab GDP dissociation inhibitor beta	Regulates the GDP/GTP exchange reaction of Rab proteins
GLYR1	Putative oxidoreductase GLYR1	Promotes KDM1B demethylase activity
GNPDA1	Glucosamine-6-phosphate isomerase 1	Conversion of D-glucosamine-6-phosphate into D-fructose-6-phosphate and ammonium
GOLM1	Golgi membrane protein 1	Unknown
GPX8	Probable glutathione peroxidase 8	Unknown
HDDC2	HD domain-containing protein 2	Unknown
HSD17B10	3-hydroxyacyl-CoA dehydrogenase type-2	Mitochondrial tRNA maturation
KIF5B	Kinesin-1 heavy chain	Distribution of mitochondria and lysosomes
KPNA2	Importin subunit alpha-1	Nuclear protein import
KPNB1	Importin subunit beta-1	Nuclear protein import
LDHB	L-lactate dehydrogenase B chain	Synthesizes lactate from pyruvate
LIMCH1	LIM and calponin homology domains-containing protein 1	Unknown
LPL	Lipoprotein lipase	Hydrolysis of triglycerides of chylomicrons and very low density lipoproteins
LRRN1	Leucine-rich repeat neuronal protein 1	Unknown
MAP4	Microtubule-associated protein 4	Promotes microtubule assembly
MARS	Methionine–tRNA ligase, cytoplasmic	Ligation of methionine to tRNA molecules
MAT2B	Methionine adenosyltransferase 2 subunit beta	Regulatory subunit of S-adenosylmethionine synthetase 2
MPI	Mannose-6-phosphate isomerase	Mannosyl transfer reactions
MRPL2	39 S ribosomal protein L2, mitochondrial	Component of the 39 S mitochondrial ribosome subunit
MYO1D	Unconventional myosin-Id	Intracellular movement
MYRIP	Rab effector MyRIP	Melanosome transport
NDUFB3	NADH dehydrogenase [ubiquinone] 1 beta subcomplex subunit 3	Mitochondrial respiratory chain NADH dehydrogenase
NHP2	H/ACA ribonucleoprotein complex subunit 2	Ribosome biogenesis
NOV	Protein NOV homolog	Cell proliferation, adhesion, differentiation
PARK7	Protein DJ-1	Oxidative stress and cell death protection
PDAP1	28 kDa heat- and acid-stable phosphoprotein	PDGFA-stimulated fibroblast growth
PLS3	Plastin-3	Actin-bundling protein of intestinal microvilli, stereocilia, fibroblast filopodia
RABGAP1	Rab GTPase-activating protein 1	Unknown
RHOT2	Mitochondrial Rho GTPase 2	Mitochondrial trafficking
RPS13	40S ribosomal protein S13	Component of the ribosomal 40S subunit
RTCB	tRNA-splicing ligase RtcB homolog	Subunit of tRNA-splicing ligase
SCPEP1	Retinoid-inducible serine carboxypeptidase	Unknown
SEC23B	Protein transport protein Sec. 23B	ER-derived vesicle transport
SLC1A5	Neutral amino acid transporter B(0)	Sodium-dependent amino acid transport
SLC7A5	Large neutral amino acids transporter small subunit 1	L-leucine transport across the blood-retinal barrier
SMARCD1	SWI/SNF-related matrix-associated actin-dependent regulator of chromatin subfamily D member 1	Chromatin remodeling
SPR	Sepiapterin reductase	Reduction of pteridine derivatives
TKFC	Triokinase/FMN cyclase	Dihydroxyacetone phosphorylation
TNKS1BP1	182 kDa tankyrase-1-binding protein	Colocalizes with chromosomes in mitosis
TPRN	Taperin	Sensory epithelial protein associated with autosomal recessive deafness.
TSPAN4	Tetraspanin-4	Cell surface glycoprotein binding to integrin
TWF2	Twinfilin-2	Actin-binding protein involved in motile and morphological processes
TXNL1	Thioredoxin-like protein 1	Active thioredoxin
TYR	Tyrosinase	Formation of pigments, melanin production from tyrosine
UBE2O	E2 ubiquitin-conjugating enzyme	Monoubiquitination of target proteins
UNC13D	Protein unc-13 homolog D	Vesicle maturation during exocytosis
USP5	Ubiquitin carboxyl-terminal hydrolase 5	Cleaves multiubiquitin polymers
VCL	Vinculin	Actin binding protein involved in cell-matrix adhesion and cell-cell adhesion
VPS25	Vacuolar protein-sorting-associated protein 25	Sorting of ubiquitinated membrane proteins during endocytosis
YAP1	Transcriptional coactivator YAP1	Critical regulatory target of the Hippo signaling pathway

The cytoskeleton-related proteins ezrin and desmoplakin, and perioxiredoxins 2 and 3, important antioxidant enzymes of the cytosol and mitochondria, respectively^33^, were upregulated during storage. Expression of vinculin and microtubule-associated protein 4 was reduced during storage. Vinculin is a membrane-associated protein that functions as a multiprotein linker to the actin cytoskeleton^34^, while microtubule-associated protein 4 is involved in crosslinking of microtubules to actin filaments^35^. The expression levels of several proteins associated with important RPE functions were specifically analyzed. The list of proteins was selected based on their known roles in visual pigment generation, phagocytosis and adhesion of RPE^36–40^. Only one of the selected proteins important for specific RPE functions had significantly changed regulation in the stored cells compared to the control (Table 3). Tyrosinase was slightly, but significantly downregulated in stored cells compared to control cells (fold change 0.8; *P* < 0.01).

**Table 3:** Effect of storage on the expression of some proteins associated with RPE-specific functions. Fold change represents changes in cells stored for three days compared to control cells.

Gene Symbol	Gene Description	Role in RPE	Fold change	P value (T test)
RPE65	Retinoid isomerohydrolase	Visual pigment regeneration	1.1	0.21
RLBP1 (CRALBP)	Retinaldehyde-binding protein 1	Visual cycle	1	0.93
TYR	Tyrosinase	Pigmentation	0.8	0.0038
PMEL	Melanocyte protein PMEL	Pigmentation	1	0.65
TYRP1	5,6-dihydroxyindole-2-carboxylic acid oxidase	Pigmentation	0.9	0.32
TYRP2	L-dopachrome tautomerase	Pigmentation	0.8	0.23
MFGE8	Lactadherin	Phagocytosis	1	0.81
ZO-1	Tight junction protein ZO-1	Tight junctions	1	0.90
OCLN	Occludin	Tight junctions	0.9	0.75
KRT18	Keratin, type I cytoskeletal 18	Cytoskeleton	0.9	0.08

## Discussion

The present study indicates that the storage viability of hRPE cells can be increased by supplementing the serum-free MEM-based storage medium containing sericin with a combination of three additional additives, while maintaining a differentiated morphology and with only slight phenotypic changes. A total of 47 individual additives were studied, including 32 combinations of the five most promising additives using a full-factorial design experiment. Herein, the five most promising storage media additives (adenosine, allopurinol, β-glycerophosphate, L-ascorbic acid and taurine) were investigated simultaneously. Compared to one-factor-at-a-time (OFAT) studies, factorial experiments have several advantages^41^. First, they require less time, material, and number of experiments, making them more cost-effective. Second, they yield better estimates of the effects of each factor because all observations are used to calculate the effect of each individual variable. Third, they reveal interactions between factors and thus permit the exploration of optimal combinations over the entire repertoire of substances. Hence, compared to OFAT studies, which vary only one factor at a time, factorial experiments simultaneously inspecting several factors are far more efficient when analyzing the effect of two or more variables.

The full-factorial experiment revealed that adenosine, allopurinol and L-ascorbic acid together provided the most desirable additive combination with regard to cell viability. This finding was controlled using CAM fluorescence measurements and validated by flow cytometry. The combined effects of these additives on hRPE storage have not been described earlier, but their individual effects on many cellular processes have been widely studied. Adenosine is a purine nucleoside which has been shown to participate in the regulation of inflammatory responses by limiting inflammatory tissue destruction^42^. Adenosine binds G protein-coupled adenosine receptors^43^, and A_3_ receptor activation has been demonstrated to protect retinal cultures against neurodegeneration^44^. Activation of the ATP receptor P2X7 is known to induce death of retinal ganglion cells, but simultaneous intravitreal injection of an A_3_ receptor agonist can prevent the P2X7-associated cell death^45^. P2X7 overactivation results in dysregulated calcium signaling and is involved in the age-related dysfunction and degeneration of RPE cells^46^. This suggests that overactive purinergic signaling may contribute to the geographic atrophy seen in dry AMD^47^. The activation of adenosine receptors and inhibition of P2X7 is considered clinically relevant for the prevention of cell death in several eye diseases, including AMD^47^. Whether the beneficial effect of adenosine on preventing P2X7-associated cell death is responsible for providing increased hRPE cell viability, or other mechanisms are at play, warrants further study.

Allopurinol is a xanthine oxidase inhibitor that reduces the production of uric acid and is being investigated for management of reperfusion injury. It has been shown to prevent postasphyxial changes in newborn pig retinas^48^ and has been successfully used in the treatment of autoimmune uveitis in an experimental setting^49^. Allopurinol administered to RPE cell cultures in high doses has been demonstrated to prevent free-radical-induced cell damage^50^. Its proposed effect on quenching free radicals might have contributed to enhancing cell viability of cultured hRPE cells during storage in the present study.

It has been established that high levels of antioxidant vitamins can significantly reduce the risk of advanced AMD and its associated vision loss in patients with intermediate or advanced AMD^51^. The addition of ascorbic acid to primary RPE cell cultures *in vitro* has been demonstrated to provide a dose-related downregulation of early-response proteins that are triggered by oxidative stress^52^. In a study using the RPE cell line ARPE-19, however, ascorbic acid was not shown to protect the cells from hydroxyl radical induced cell death^53^. Yet other studies have shown that ascorbic acid supplementation can protect RPE cells from hypoxic damage^54^ and reduce vision cell loss from damaging light^55^. However, the latter effect might be attributable to ascorbic acid preventing excessive shedding of rod outer segments upon light exposure^56^. The effect of ascorbic acid in the present study might be similar to that of allopurinol in that it reduces the oxidative stress burden.

Our research group recently demonstrated that sericin induces melanogenesis of hRPE cells through activation of the NF-κB pathway^21^. Sericin has been shown to inhibit tyrosinase^57^, and proteomic analysis in the present study confirmed that tyrosinase expression is slightly reduced in cells stored in the optimal additive combination in the presence of sericin. The expression of other pigment-related proteins (premelanosome protein 17, tyrosinase related protein 1 and tyrosinase related protein 2) was maintained during storage using the optimal additive combination. Tyrosinase is the main rate-limiting melanogenesis enzyme, catalyzing the formation of dihydroxyphenylalanine (L-DOPA) from L-tyrosine^58^. However, light microscopy and TEM demonstrated the presence of melanized cells and melanosomes in stored cell cultures. While phase contrast and transmission electron microscopy can determine the presence of melanosomes, these are not satisfactory methods by which to objectively determine the level of pigmentation. Future studies warrant the use of other methods, i.e. spectrophotometry or modified scanning devices as demonstrated by Lane *et al*.^59^.

In a study by Vugler *et al*.^60^ investigating RPE cells differentiated from human embryonal stem cells (HESC-RPE), a larger number of stage 4 melanosomes were displayed; however, these cells were of a different origin and were cultured under very different conditions than used in the present study. For instance, the HESC-RPE were cultured on Matrigel for five weeks. Polarization was evident with basally oriented nuclei like in our cells, but apical microvilli were more developed in this study than is shown in our cultures. Both the cell source and culture length might be of essence in order to further enhance differentiation^61–63^. Ultrastructure is presented in great detail in a study by Carr *et al*.^64^, who demonstrated that co-culture of HESC-RPE with human retina leads to maturation-associated morphological alterations. Herein, the presence of melanosomes, tight junctions and microvilli is demonstrated. Similar findings are made in control cells and cells stored in the optimal additive combination in this study (Fig. 6).

Pyruvate has been shown to induce pigmentation of ARPE-19 cells cultured in DMEM with high glucose^61^. In our study, the basic storage medium was supplemented with pyruvate, which might have contributed further to the increased pigmentation demonstrated in both the current and earlier studies by our research group. Although several culture protocols using hESCs or iPSCs have successfully produced differentiated and pigmented RPE cells, they are usually more time-consuming^62,63^. The use of sericin might contribute in shortening the culture period. The focus on the differentiation process is critical, as its efficiency is considered crucial to the economic feasibility of regenerative therapy using RPE cells^59^.

The expression of the tight junction protein ZO-1 was maintained during storage, as demonstrated by proteomics analysis and transmission electron microscopy. Cultured cells established the classic hexagonal distribution of mature hRPE monolayers. The RPE, being a polarized monolayer, is dependent on functional intercellular tight junctions to maintain high transepithelial resistance, secure cellular barrier function and regulate paracellular permeability^65–68^. Hence, the present study confirms earlier findings, but still indicates that hRPE cells can retain features of a mature phenotype when stored in the optimal additive combination.

The cytoskeleton-related proteins ezrin and desmoplakin were upregulated during storage. Ezrin is a cortical cytoskeleton protein which localizes to epithelial microvilli^69^. Loss of ezrin function as demonstrated in ezrin knockout mice leads to substantial reduction in RPE apical microvilli and retarded photoreceptor development^69^. Desmoplakin is necessary for the anchoring of keratin at cell-cell contacts^70^, and thus important for the regulation of desmosomal adhesion strength^71^. It functions as a tumor suppressor^72^, and a decrease in desmosomal protein expression is associated with poor prognosis in several cancers^73–75^. Loss-of-function mutations in desmosomal proteins have been associated with clinical syndromes involving the skin, heart, hair and immune system^76–79^. Upregulation of these proteins during storage might indicate that stored cells maintain robust cytoskeletal functions.

Third passage hRPE cells were employed in this study. The increased tendency of epithelial to mesenchymal transition with increasing passages of RPE cells has been demonstrated by Grisanti *et al*. They showed a large disparity between passage 2 RPE and passage 10 RPE, where cells of the higher passages transdifferentiate and lose differentiated RPE properties^80^. While there is a wide consensus regarding the advantages of using early-passage RPE cells to avoid this phenomenon in culture, an exact passage number has not been defined. In a study by Ganti *et al*. investigating vitreous modulation of gene expression in low-passage hRPE, cells from passages 3–6 were termed “early-passage”^81^. Based on the observed benefits of early passage cell lines, we selected third passage hRPE cells for this study.

In conclusion, the current study demonstrates that the storage medium additive combination of sericin, adenosine, allopurinol and L-ascorbic acid successfully maintains hRPE cell viability during storage while preserving the characteristic hRPE morphology and proteome. The effects of the individual additives are not thoroughly understood, but previous research points to free radical scavenging mechanisms as possible explanations for these findings.

Future studies should investigate the effect of increased storage duration on hRPE cells in the optimal combination medium, and ideally expand the scope to RPE derived from different sources, including primary human stem cells and induced pluripotent stem cells. This could provide valuable knowledge when establishing a storage protocol for clinical use.

## Methods

### Supplies

Primary hRPE and complete epithelial cell medium (EpiCM) were purchased from ScienCell Research Laboratories (San Diego, CA). Dulbecco’s Modified Eagle’s Medium (high glucose, with pyruvate; hereafter named DMEM), Minimal Essential Medium, heat-inactivated fetal bovine serum (FBS), N1 growth supplement, taurine, triiodothyronine, non-essential amino acids, glutamine-penicillin-streptomycin, hydrocortisone, propidium iodide (PI), phosphate-buffered saline (PBS) and 4′,6-diamidino-2-phenylindole (DAPI) were obtained from Sigma Aldrich (St Louis, MO). Nunclon Δ surface plates, pipettes and other routine plastics were purchased from VWR International (West Chester, PA). The calcein-acetoxymethyl ester (CAM)/ethidium homodimer 1 (EH-1) viability kit was purchased from Invitrogen. The 47 additives used in the study are listed in Supplementary Information, Table S1.

### Culture and Preparation of Cells

Third passage hRPE were seeded (20,000 cells/cm^2^) in complete EpiCM on 96-well Nunclon Δ surface plates and cultured under routine conditions of 95% air and 5% CO_2_ at 37 °C. After two days, EpiCM was replaced with modified DMEM (hereafter named «differentiation medium») containing 4.5 g/L glucose, pyruvate, 1% sericin, and 1% penicillin-streptomycin. Cells were then cultured for 14 days in differentiation medium until pigmentation, as demonstrated in an earlier study^21^. The culture medium was changed every two or three days.

### Storage of hRPE cells

Cells were cultured in the differentiation medium for 14 days, until cells were confluent and >20% of cells were pigmented as visually determined by phase contrast microscopy. The differentiation medium was then removed and the cultures were rinsed with PBS before addition of storage medium. The storage medium consisted of 0.3 mL MEM, 25 mM 2-[4-(2-hydroxyethyl)piperazin-1-yl]ethanesulfonic acid (HEPES), 22.3 mM sodium bicarbonate, 50 µg/mL gentamycin, and 1% sericin. A total of 46 different additives were individually supplemented to the storage medium and sterile-filtered (pore size 0.2 μm) before being added to the culture wells (N = 3) using a Biomek® 4000 Laboratory Automation Workstation (Beckman Coulter, Inc., Brea, CA). All cultures were stored at 4 °C for ten days, without change or addition of storage medium. The storage containers were custom-built as reported elsewhere^82^. pH measurements of the storage medium were performed using pH indicator paper.

### Viability Analysis using Quantitative Immunofluorescence

Cell viability was analyzed after 10 days of storage by incubating the stored cells with PBS containing 1.0 μM CAM and 1.0 μM EH-1 for 30 min. CAM is enzymatically cleaved into the green fluorescent calcein inside living cells. EH-1 is a membrane-impermeable dye that binds to DNA of dead cells. Area of fluorescence was calculated for all additive groups using epifluorescence microscopy and custom-made macros with ImageJ software (National Institutes of Health, Bethesda, MD). In detail, photomicrographs were captured at 200x magnification at five predetermined locations in each culture well using a Nikon Eclipse Ti fluorescence microscope (Nikon Instruments, Tokyo, Japan) with a DS-Qi1 black-and-white camera (Nikon Instruments) and a motorized microscope stage. Identical exposure length and gain were used for all compared groups, while keeping the image brightness within the camera’s dynamic range.

**Figure 9:**
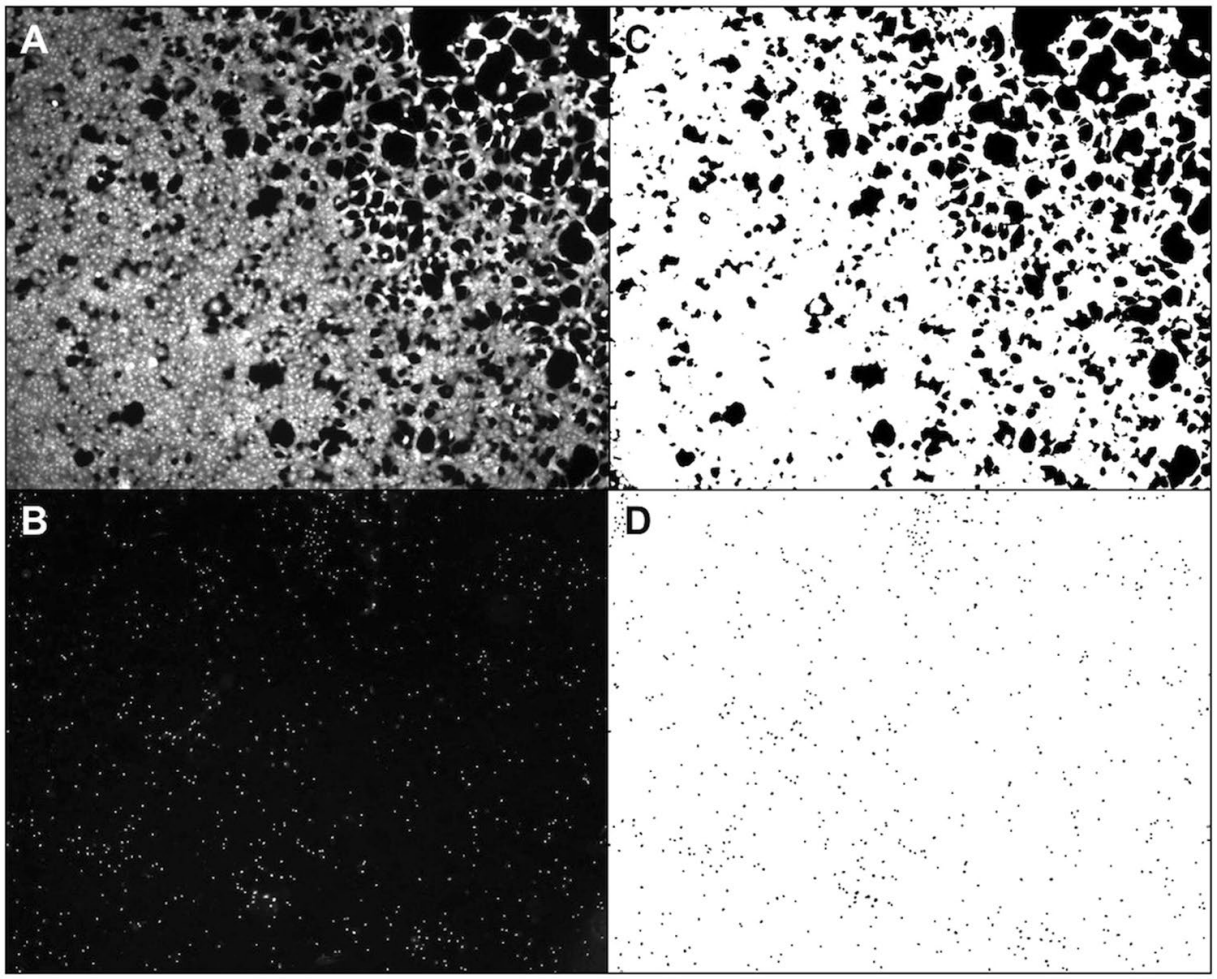
Quantification of viable cells. Following storage, hRPE cells were stained with calcein-acetoxymethyl ester (CAM) (**A**) to visualize viable cells and ethidium homodimer-1 (EH-1) (**B**) to identify dead cells. Images of CAM-stained (**C**) or EH-1-stained (**D**) cells were segmented by ImageJ based on the fluorescence intensity. To compare the amount of live and dead cells between groups, ImageJ quantified the area (white) of the viable cells (**C**) and counted the number of particles that represented dead cell nuclei (**D**).

ImageJ software was used to subtract unevenly transmitted light from all 16-bit photomicrographs using the “Subtract Background” -command. All photomicrographs were converted to binary photos before the “Area Fraction” -command was used to measure the culture well area covered by CAM-stained cells. The number of EH-1 stained nuclei was automatically counted using the “Analyze Particles” -command (Fig. 9).

### Factorial Design

A factorial design experiment is a complex statistical design offering the possibility to study more than one factor at a time by creating a simulation of combined factor effects. Factorial design using Design-Expert (Stat-Ease, Inc., Minneapolis, MN) was employed to identify the most promising combination of storage medium additives. The five best additives from the viability analysis were included as independent variables (adenosine, allopurinol, β-glycerophosphate, L-ascorbic acid and taurine), with area of CAM fluorescence and the number of dead cells as the two dependent variables. The combined results of two end points were studied. However, the «Importance» tool was employed to set relative priorities for the two variables. The importance of viability (CAM fluorescence area) was emphasized over cell death (number of dead cells). The two-level full-factorial design included replicates of all 32 possible combinations of the five additives. Data were fitted to a full quadratic model. ANOVA was used to calculate the adjusted significance of both models (viability and death) in Design-Expert (*P* = 0.0047 and *P* = 0.036, respectively).

### Flow Cytometry

Flow cytometry was used to validate the viability results. Cells were cultured in T25 cell culture flasks following the aforementioned protocol. Control cells (N = 3) and cells stored in the optimal additive combination (1% sericin, 5 mmol/L adenosine, 50 μg/mL L-ascorbic acid and 1 mM allopurinol) (N = 3) for three days were compared. Propidium iodide (PI), which binds to double-stranded DNA of dead cells, was added to the culture medium of both culture groups at a concentration of 2.5 μg/300 μL sample and cells were returned to the incubator for 15 minutes. Cells were then rinsed with PBS, trypsinized for 2–3 minutes, then washed and re-suspended in ice-cold HBSS +4% FBS. Samples were kept on ice and analyzed using the BD Accuri C6 bench top flow cytometer. PI is excited by the 588 nm laser and is detected in filter 616//23 (FL3).

### Transmission Electron Microscopy

Both unstored cultures and samples of hRPE stored for three days in MEM storage medium with the optimal additive combination (1% sericin, 5 mmol/L adenosine, 50 μg/mL L-ascorbic acid and 1 mM allopurinol) were processed for transmission electron microscopy (TEM) analysis as described earlier^83^. In essence, a Leica Ultracut Ultramicrotome (Leixa, Wetzlar, Germany) was used to cut ultrathin sections, which were examined using a CM120 transmission electron microscope (Philips, Amsterdam, the Netherlands).

### Statistical Analysis

Statistical analysis was performed using IBM SPSS Statistics for Macintosh version 22.0 (IBM Corp, Armonk, NY). A one-way analysis of variance with Tukey’s post-hoc comparisons was used for statistical evaluation of the viability results. The Student’s t-test was used to compare two groups. *P* values below 0.05 were considered significant.

### Proteomics

The proteome of hRPE cells stored in the optimal storage medium combination was analyzed and compared to control cells that had not been stored. The proteome analyses were performed as previously described^84^. Briefly, the proteins of cell lysates were digested in-solution with trypsin. The generated peptides were analyzed by LC-MS using a nano-UHPLC connected to a Q Exactive mass spectrometer. Proteins were identified using the Mascot search engine and Scaffold software (version Scaffold_4.7.3, Proteome Software Inc., Portland, OR) was used for further data analysis and label-free quantification. Scaffold was used to validate MS/MS based peptide and protein identifications. Peptide identifications were accepted if they could be established at greater than 95.0% probability by the Peptide Prophet algorithm^85^ with Scaffold delta-mass correction. Protein identifications were accepted if they could be established at greater than 99.0% probability and contained at least 2 identified peptides. Protein probabilities were assigned by the Protein Prophet algorithm^86^. Proteins that contained similar peptides and could not be differentiated based on MS/MS analysis alone were grouped to satisfy the principles of parsimony. Distribution of protein functions in hRPE before and after storage was determined using Scaffold software with annotations downloaded from the NCBI web database.

### Data availability

The datasets generated and analyzed during the current study are available from the corresponding author on request.

## Electronic supplementary material

Supplementary Information
