# Supplementary material for: Improvement of Storage Medium for Cultured Human Retinal Pigment Epithelial Cells Using Factorial Design
Source: Sci Rep. 2018 Apr 9;8:5688. doi: 10.1038/s41598-018-24121-8 (PMC5890280; doi:10.1038/s41598-018-24121-8)
Supplement: Supplementary file 1 — Supplementary Information [file 41598_2018_24121_MOESM1_ESM.docx]

**Improvement of Storage Medium for Cultured Human Retinal Pigment Epithelial Cells Using Factorial Design**

L. Pasovic*^1,2^, T. P. Utheim^1,3^, S. Reppe^1^, A. Z. Khan^1,2^, C. J. Jackson^1^, B. Thiede^4^, J. P. Berg^1^, E. B. Messelt^3^ & J. R. Eidet^5^

^1^Department of Medical Biochemistry, Oslo University Hospital, Oslo, Norway;

^2^Institute of Clinical Medicine, University of Oslo, Oslo, Norway;

^3^Department of Oral Biology, Faculty of Dentistry, University of Oslo, Oslo, Norway;

^4^Department of Biosciences, University of Oslo, Oslo, Norway;

^5^Department of Ophthalmology, Oslo University Hospital, Oslo, Norway

*Correspondence should be addressed to L.P. (larapasovic@gmail.com).

**Supplementary Information**

**Table S1.** **Additives used in the present study.**

|  | **Additive** | **Concentration** | **Supplier** |
| --- | --- | --- | --- |
| 1 | Adenosine | 5 mM | Sigma |
| 2 | Allopurinol | 1 mM | Sigma |
| 3 | Arachidonic acid | 1 mg/L | Sigma |
| 4 | β-glycerophosphate | 10 mM | Sigma |
| 5 | BSA | 1 % | Sigma |
| 6 | Capsazepine | 10 μM | Sigma |
| 7 | Carnosine | 20-50 mM | Sigma |
| 8 | Corn starch | 5 mg/mL | Sigma |
| 9 | Creatine | 1 mM | Sigma |
| 10 | DADLE | 1 mM | Sigma |
| 11 | Deferroxamine mesylate | 10 μmol/L | Sigma |
| 12 | DHA | 50 nM | Sigma |
| 13 | Galactose | 10 mg/mL | Koch Light Laboratories |
| 14 | Genistein | 30 μM | Sigma |
| 15 | Gluthatione | 3 mM | Sigma |
| 16 | Glycerol | 10 mg/mL | Sigma |
| 17 | HGF | 100 ng/mL | Sigma |
| 18 | Hydrocortisone | 3 ng/mL | Sigma |
| 19 | IGF1 | 100 ng/mL | Sigma |
| 20 | Imidazole | 200 mM | Sigma |
| 21 | Insulin | 5 μg/mL | Sigma |
| 22 | JSH-23 | 5 μM | Sigma |
| 23 | Kolliphor 188 | 0.05 % | Sigma |
| 24 | L-ascorbic acid | 50 μg/mL | Sigma |
| 25 | Lactic acid | 5 mg/mL | Sigma |
| 26 | Lactobionate | 80 mM | Sigma |
| 27 | Lactose | 30 mM | Sigma |
| 28 | Laevulose | 10 mg/mL | Koch Light Laboratories |
| 29 | Leukemia Inhibitory Factor | 10 ng/mL | Sigma |
| 30 | LiCl | 1 mM | Merck |
| 31 | Linoleic acid | 1 mg/L | Sigma |
| 32 | Memantine | 30 μM | Sigma |
| 33 | Na Pyruvate | 10 mM | Sigma |
| 34 | Oleic acid | 1 mg/L | Sigma |
| 35 | PEDF | 4 nM | Sigma |
| 36 | Protease inhibitor coctail | 0.25% | Sigma |
| 37 | Quercetin | 10 μM | Sigma |
| 38 | Raffinose | 30 mM | Sigma |
| 39 | Resveratrol | 30 μM | Sigma |
| 40 | Riboflavin | 50 μM | Sigma |
| 41 | Sericin (control) | 1 % | Sigma |
| 42 | Sorbitol | 10 mg/mL | Sigma |
| 43 | Sucrose | 10 mg/mL | BDH |
| 44 | Taurine | 20 mM | Sigma |
| 45 | Triamincinolone acetonide | 0.1 mg/mL | Sigma |
| 46 | Triiodothyronine | 0.013 μg/L | Sigma |
| 47 | Urea | 10 mg/mL | Merck |

Abbreviations: BSA: bovine serum albumin; DADLE: [D-Ala^2^, D-Leu^5^]-Enkephalin; DHA: docosahexaenoic acid; HGF: hepatocyte growth factor; IGF1: insulin-like growth factor 1; JSH-23: 4-methyl-1-N-(3-phenylpropyl)benzene-1,2-diamine; PEDF: pigment epithelium-derived factor.
